# Pelvic Floor Disorders 6 Months after Attempted Operative Vaginal Delivery According to the Fetal Head Station: A Prospective Cohort Study

**DOI:** 10.1371/journal.pone.0168591

**Published:** 2016-12-16

**Authors:** Guillaume Ducarme, Jean-François Hamel, Stéphanie Brun, Hugo Madar, Benjamin Merlot, Loïc Sentilhes

**Affiliations:** 1 Department of Obstetrics and Gynecology, Centre Hospitalier Departemental, La Roche sur Yon, France; 2 Clinical Research Center, Angers University Hospital, Angers, France; 3 Department of Obstetrics and Gynecology, Bordeaux University Hospital, Bordeaux, France; Oslo universitetssykehus Ulleval, NORWAY

## Abstract

**Objective:**

To evaluate the effect of the fetal head station at attempted operative vaginal delivery (aOVD), and specifically midpelvic or low aOVD, on urinary incontinence (UI), anal incontinence (AI), and perineal pain at 6 months.

**Design:**

Prospective cohort study.

**Setting:**

1941 women with singleton term fetuses in vertex presentation with midpelvic or low aOVD between 2008 and 2013 in a tertiary care university hospital.

**Methods:**

Symptoms of urinary incontinence (UI) using the Bristol Female Lower Urinary Tract Symptoms questionnaire, and symptoms of anal incontinence (AI) severity using Fecal Incontinence Severity Index (FISI) were assessed 6 months after aOVD. We measured the association between midpelvic or low aOVD and symptoms of UI, AI, and perineal pain at 6 months using multiple regression and adjusting for demographics, and risk factors of UI and AI, with adjusted odds ratios (aORs) and 95% confidence intervals (95% CI).

**Results:**

The study included 907 women (46.7%) who responded to the questionnaire; 18.4% (167/907) had midpelvic aOVD, and 81.6% (740/907) low; and none of women with symptoms of UI (26.6%, and 22.4%, respectively; p = 0.31), AI (15.9%, and 21.8%; p = 0.09), the FISI score, and perineal pain (17.2%, and 12.7%; p = 0.14) differed significantly between groups. The same was true for stress, urge, and mixed-type UI, severe UI and difficulty voiding. Compared with low pelvic aOVD, the aORs for symptoms of UI in midpelvic aOVD were 0.70 (0.46–1.05) and AI 1.42 (0.85–2.39). Third- and fourth-degree tears were a major risk factor of symptoms of UI (aOR 3.08, 95% CI 1.35–7.00) and AI (aOR 3.47, 95% CI 1.43–8.39).

**Conclusion:**

Neither symptoms of urinary nor anal incontinence differed at 6 months among women who had midpelvic and low pelvic aOVD. These findings are reassuring and need further studies at long-term to confirm these short-term data.

## Introduction

Although most women want a spontaneous vaginal delivery, a non-negligible proportion of them have a labor that fails to progress during the second stage of labor and can thus leave the fetus at midpelvic station. Obstetricians are then faced with a choice between a potentially difficult operative vaginal delivery (OVD) and cesarean delivery at full dilatation with a fetus in midpelvis, each with its own immediate and long-term maternal and neonatal risks. This situation seems to present the perfect setting for a randomised trial, which would probably fail to achieve due to a neither feasible nor ethical recruitment in the antenatal period. Furthermore, when the fetus is at low pelvic station, OVD is not discussed for maternal or fetal indication, and must be preferred to caesarean delivery [1]. Our previous report that midpelvic attempted operative vaginal delivery (aOVD) was not associated with a higher rate of severe short-term maternal and neonatal morbidity than attempted low pelvic delivery suggests that midpelvic aOVD is an appropriate option in selected women [2].

However, if its mid- and long-term maternal or neonatal outcome is worse than that for low pelvic OVD, cesarean delivery might be preferred over an OVD when the fetus is at midpelvis. Many studies have assessed pelvic floor symptoms, urinary incontinence (UI), anal incontinence (AI) and perineal pain during the postpartum period according to the mode of delivery (spontaneous vaginal delivery, OVD, caesarean), or complications of pregnancy or delivery [3–13]. Findings vary widely. These discrepancies may be explained by methodological differences (retrospective, prospective or ambispective designs, sample size, or time during the postpartum period chosen for evaluation). To our knowledge, no study has specifically evaluated postpartum pelvic floor symptoms after an aOVD according to the fetal head station. In a prospective based cohort study, we aimed to compare pelvic floor symptoms after aOVD for midpelvic and for low aOVDs at 6 months, and to analyse the risk factors of symptoms of UI and AI on this population.

## Material and Methods

### Study sample

The study protocol was approved by the Institutional Review Board at the Angers University Hospital, France, on November 2008 (Study ID: 2008) [2]. This study was conducted in accordance with French law. All subjects were told about the study and were given oral information. Verbal consent was provided to the medical team in charge of the study. Our study was done using data from a prospective based cohort study including 1,941 women with live singleton term fetuses in vertex presentation who underwent an aOVD at midpelvic or low from December 2008 through October 2013 in a tertiary care university hospital with more than 4,000 annual deliveries [2]. Pre-specified study design was to analyse short-term maternal and neonatal morbidity according to the fetal head station (midpelvic or low aOVD) [2], and to prospectively analyse long-term maternal complication (pelvic floors disorders, sexual dysfunction, maternal postpartum depressive symptoms at 6 months and 5 years), and children development at 5 years, according to the fetal head station, and specifically in midpelvic or low pelvic aOVD. As described in detail previously [2], this study included all women with an aOVD, defined by the placement of at least one blade for forceps or spatula or a vacuum, regardless of its success (i.e., whether delivery was finally vaginal or caesarean), and a live singleton pregnancy in vertex presentation at term (equal to or later than 37 weeks of gestation) [2]. Exclusion criteria were multiple gestations, fetal growth restriction (FGR), defined as <10^th^ centile for gestational age on Hadlock curves [14,15], a known congenital anomaly, vaginal breech delivery, and the absence of fetal station information according to the American College of Obstetricians and Gynaecologists (ACOG) classification [16]. Specifically, station was defined by the level of the leading bony point of the fetal head in centimetres at or below the level of maternal ischial spines (0 and +1 = midpelvic; +2 and +3 = low). Two cohorts of women were assessed separately: those with a midpelvic aOVD (N = 391; 20.6%), and a lowpelvic aOVD (N = 1,550; 79.4%).

### Measures

Information about pelvic floor disorders was obtained from a questionnaire sent 6 months after delivery. A second mailing was sent to the women from whom we received no response. A questionnaire asked about postpartum pelvic floor exercises and pelvic floor symptoms during the preceding 4 weeks. Those women who answered yes to the entry question “Do you have involuntary loss of urine?” were considered to have symptoms of UI and were then asked further questions from the French version of a validated questionnaire *Bristol Female Lower Urinary Tract Symptoms* (BFLUTS) [8,17,18], about the frequency, amount, and circumstances of leakage and if incontinence was a problem for them. Stress urinary incontinence (SUI) was assessed by responses to the question "Does urine leak when you are physically active, cough or sneeze?" Possible responses were as follows: never, occasionally, sometimes, often or all the time. Occasionally was defined as less than one-third of the time; sometimes as between one and two-thirds of the time; and often as more than two-thirds of the time. Women who answered “often” or “all the time” were considered to have severe SUI [8,17,18]. Urge incontinence was assessed by any positive response to "Does urine leak before you go to the toilet?", and mixed incontinence by a positive response to both of previous questions. Voiding difficulty was assessed by response to the question "Do you have difficulties in emptying your bladder?" [8,17].

As previously reported [8], perineal pain was evaluated through the question: “Do you have chronic perineal pain (perineum designates the skin and muscle around the vaginal and anal outlets)?”. This question was dichotomous with two possible answers: “yes” and “no” [8]. Episiotomy complications were evaluated through the question: "Do you have any complications concerning your episiotomy (hematoma, abscess, scar disunion, surgery)?" They were defined by the existence of at least one of the following criteria: hematoma, abscess, scar disunion, or required surgery for episiotomy.

Anal incontinence was defined by yes (versus no) response to “Do you have involuntary loss of flatus or stool?” [8]. The severity of symptoms of AI was assessed with the French version of the *American Society of Colon and Rectal Surgeon’s Fecal Incontinence Severity Index* (FISI) [19,20]. The FISI is based on [(type of incontinence) x (frequency matrix)]. The matrix includes four types of leakage commonly found in the faecal-incontinent population (gas, mucus, and liquid and solid stool) and five frequencies classified as involuntary loss 2 or more times a day, once a day, 2 or more times a week, once a week, 1–3 times a month, or never. To create the FISI, responses for each of the four items were summed, with a higher FISI indicating greater perceived symptoms. The questionnaire used for the study is available (S1 Questionnaire).

### Statistical analysis

Continuous data were described by their means ± standard deviations and compared by t-tests (or Mann-Whitney tests when appropriate); categorical data were described by percentages and compared by chi-square tests (or Fisher exact tests when appropriate). Univariate and multivariate logistic regression were used to study the association between UI or AI at 6 months (considered as a dichotomous variable) and aOVD classification. Univariate and multivariable linear regression analysis were conducted to study the association between AI severity (FISI index) at 6 months (considered as a continuous variable) and aOVD classification. Multivariate models were built with a stepwise procedure based on the Akaike criterion [21,22], and the covariate "fetal head station" was systematically forced into the models. Confounders associated in univariate analysis at a 0.2 level were included in this stepwise procedure. To check the fit of the multivariate models, we studied the studentized residuals. Wald tests were performed for testing the significance of the covariates included in the models. STATA 13.1 software (StataCorp, College Station, TX, USA) was used for all analyses. Statistical significance was defined as a *P* value < 0.05.

## Results

**Fig 1:**
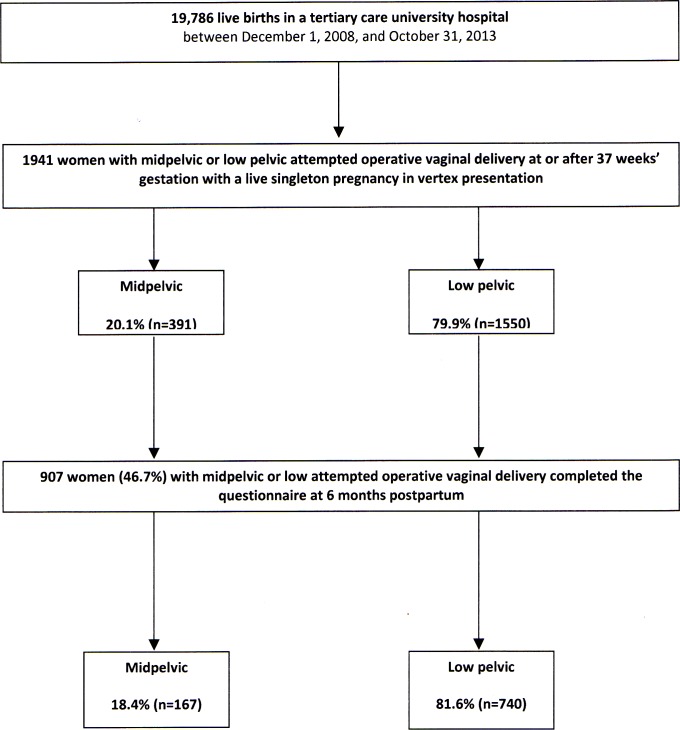
Cohort flowchart.

Six months after delivery, 907 women (46.7%) of the 1941 deliveries with an aOVD completed the questionnaire: 18.4% (N = 167) had been midpelvic, and 81.6% (N = 740) low attempted deliveries (Fig 1). The main differences between respondents and non-respondents were maternal age at delivery (29.0 ± 4.8 years compared to 27.6 ± 5.2 years; p<0.001), marital status (96.1% married or living with a partner compared to 91.9%; p<0.001), severe neonatal morbidity (9.6% compared to 12.8%; p = 0.03), rates of NICU transfer (5.0% compared to 8.4%; p = 0.003), and prolonged hospitalisation (>24 hours) in NICU (4.2% compared to 7.4%; p = 0.003). The fetal head station did not differ significantly between respondents and non-respondents (S1 Table).

Table 1 summarizes the characteristics of the women and their labour, and the maternal and neonatal outcomes among respondents. Among women with aOVD, the prevalence of symptoms of UI at 6 months was 21.6% (N = 196), the prevalence of symptoms of AI 19.0% (n = 172), and 113 women (12.5%) reported perineal pain. No significant differences were observed for symptoms of UI (26.6% for midpelvic, and 22.4% for low deliveries; p = 0.31), symptoms of AI (15.9%, and 21.8%, respectively; p = 0.09), and perineal pain (17.2%, and 12.7%; p = 0.14) between these groups. The mean FISI scores in women with AI were low and did not differ between the groups (11.9±6.3 *vs*. 11.8±6.8, respectively; p = 0.93), nor did the prevalence of stress, urge, mixed-type incontinence, severe urinary incontinence or difficulty voiding (Table 1). Women with symptoms of UI were compared to women without symptoms of UI, and women with symptoms of AI compared to women without symptoms of AI (Table 2). These groups differed according to maternal age, third/fourth degree perineal tears, perineal pain, and breast-feeding, but not according to head station at aOVD (Table 2). Finally, episiotomy was not also significantly associated with any of the pelvic floor disorders considered at 6 months (Table 2).

**Table 1:** Characteristics of mothers and their labor and maternal and neonatal outcomes in respondents, according to the ACOG classification.

	Mid (N = 167)	Low (N = 740)	*P* value
**Maternal and labor characteristics**			
Maternal age, (years)^1^	29.2 ± 5.3	29.0 ± 4.7	0.59*
Geographic origin			0.34**
Europe, n (%)	156 (93.4)	699 (94.5)	
Sub-Saharan Africa, n (%)	5 (3.0)	8 (1.1)	
North Africa, n (%)	1 (0.6)	6 (0.8)	
Asia, n (%)	2 (1.2)	17 (2.3)	
Other, n (%)	3 (1.8)	10 (1.3)	
Married or living with a partner, n (%)	158 (95.8)	709 (96.2)	0.79**
Nulliparity, n (%)	118 (70.7)	564 (76.2)	0.13**
Previous cesarean delivery, n (%)	20 (42.6)	66 (37.5)	0.52**
Previous 3^rd^ or 4^th^-degree perineal lacerations, n (%)	0	1 (0.6)	0.60***
Previous depression, n (%)	5 (3.0)	38 (5.1)	0.25**
BMI before pregnancy (kg/m^2^)^1^	22.8 ± 4.1	22.7 ± 3.9	0.72*
Gestational weight gain (kg) ^1^	13.8 ± 4.5	13.3 ± 4.4	0.23*
Antenatal suspicion of macrosomia ^2^, n (%)	18 (10.8)	48 (6.5)	0.05**
Gestational age at delivery (weeks) ^1^	39.5 ± 1.5	39.4 ± 1.4	0.63*
Induced labor, n (%)	30 (18.0)	125 (16.9)	0.74**
Length of labor (min) ^1^	395.7 ± 179.1	388.4 ± 165.0	0.61*
Length of 2^nd^ stage (min) ^1^	103.3 ± 73.8	108.8 ± 67.3	0.35*
Active phase of 2^nd^ stage > 30 min, n (%)	51 (30.5)	267 (36.1)	0.28**
Dose of oxytocin (mUI) ^1^	1976.3 ± 2228.8	1620.9 ± 2084.8	0.05*
Epidural analgesia, n (%)	164 (98.2)	698 (94.5)	0.04**
Manual rotation, n (%)	30 (18.0)	81 (11.0)	0.01**
Persistent occiput			0.05**
Anterior, n (%)	139 (83.2)	662 (89.8)	
Posterior, n (%)	21 (12.6)	56 (7.6)	
Transverse, n (%)	7 (4.2)	19 (2.6)	
Indications for OVD			0.02**
Non reassuring FHR only, n (%)	86 (51.5)	301 (40.7)	
Arrested progress only, n (%)	51 (30.5)	313 (42.3)	
Non reassuring FHR and arrested progress, n (%)	30 (18.0)	129 (17.4)	
OVD in operating room, n (%)	12 (7.2)	4 (0.5)	<0.001***
Provider attending delivery			<0.001**
Senior obstetrician, n (%)	88 (54.7)	159 (21.7)	
Resident, n (%)	73 (45.3)	573 (78.3)	
Instrument type			
Vacuum, n (%)	13 (7.8)	237 (32.0)	<0.001**
Forceps, n (%)	20 (12.0)	38 (5.1)	<0.001***
Spatula, n (%)	140 (84.3)	485 (65.5)	<0.001**
Sequential use of instrument, n (%)	6 (3.6)	21 (2.8)	0.59***
**Maternal outcome**			
Cesarean section after failed OVD, n (%)	12 (7.2)	4 (0.5)	<0.001***
Episiotomy, n (%)	144 (87.3)	652 (88.1)	0.77**
3^rd^ or 4^th^-degree perineal lacerations, n (%)	3 (1.8)	25 (3.4)	0.30***
Perineal hematomas, n (%)	0	1 (0.1)	0.63***
Abscesses/hematoma required surgery, n (%)	1 (0.7)	3 (0.4)	0.69***
Postpartum hemorrhage (PPH), n (%)	35 (20.9)	128 (17.3)	0.27**
Severe PPH (blood loss>1500mL), n (%)	5 (3.0)	16 (2.2)	0.52***
Second-line therapies^3^, n (%)	1 (1.0)	1 (0.2)	0.25***
Blood transfusion, n (%)	7 (4.2)	12 (1.6)	0.04***
Infections^4^, n (%)	1 (0.7)	1 (0.1)	0.25***
Thromboembolic events, n (%)	0	2 (0.3)	0.50***
Maternal hospitalization in intensive care unit, n (%)	0	0	-
Severe maternal morbidity^5^, n (%)	13 (7.8)	64 (8.7)	0.71**
**Neonatal outcome**			
Birth weight≥4000 g, n (%)	12 (7.2)	38 (5.1)	0.30**
5-min Apgar score<7, n (%)	1 (0.6)	5 (0.7)	0.91***
pH<7.00, n (%)	4 (2.5)	10 (1.4)	0.32***
Transfer to NICU, n (%)	9 (5.4)	36 (4.9)	0.78**
NICU hospitalisation>24 h, n (%)	9 (5.4)	29 (3.9)	0.39**
Respiratory distress syndrome, n (%)	8 (4.8)	30 (4.1)	0.66**
Neonatal trauma^6^, n (%)	3 (1.8)	3 (0.4)	0.05***
Shoulder dystocia, n (%)	7 (4.4)	15 (2.0)	0.08***
Need for resuscitation or intubation, n (%)	0	8 (1.1)	0.18***
Severe neonatal morbidity^7^, n (%)	25 (15.0)	62 (8.4)	0.01**
**Registered variables at 6 months postpartum**			
Urinary incontinence, n (%)	41 (26.6)	155 (22.4)	0.31**
Stress urinary incontinence, n (%)	5 (12.2)	16 (10.5)	0.51***
Urge urinary incontinence, n (%)	13 (31.7)	52 (33.3)	0.72**
Mixed urinary incontinence, n (%)	25 (15.0)	91 (12.3)	0.35**
Difficulty voiding, n (%)	13 (29.6)	50 (31.8)	0.63**
Severe urinary incontinence, n (%)	0	2 (1.3)	0.67***
Anal incontinence, n (%)	24 (15.9)	148 (21.8)	0.09**
FISI score^1^	11.9 ± 6.3	11.8 ± 6.8	0.93*
Perineal pain, n (%)	26 (17.2)	87 (12.7)	0.14**
Breastfeeding, n (%)	95 (93.1)	470 (93.3)	0.63**
Episiotomy complications^8^, n (%)	54 (38.9)	245 (37.7)	0.80**
Pelvic floor muscle training, n (%)	119 (77.8)	544 (79.1)	0.72**

^1^ Values are given as mean ± standard deviation.

^2^ Antenatal suspicion of macrosomia: fundal height measurement at delivery > 37cm and/or ultrasonographic fetal abdominal circumference > 90^th^ p. for gestational age and sex on Hadlock curves [14].

^3^ Second-line therapies were uterine compression sutures, uterine artery embolization, and peripartum hysterectomy for management of massive primary postpartum hemorrhage after failure of uterine massage and uterotonic agents to stop bleeding [2].

^4^ Infections were defined by the existence of at least one of the following criteria: endometritis, episiotomy infection and wound infection needed surgery [2].

^5^ Severe maternal morbidity was defined by the existence of at least one of the following criteria: third or fourth-degree perineal lacerations, perineal hematomas, cervical laceration, extension of uterine incision at cesarean section, PPH>1500 mL, surgical haemostatic procedure, uterine artery embolization, blood transfusion, infections (endometritis, episiotomy infection, wound infection needed surgery), thromboembolic events (deep vein thrombophlebitis and pulmonary embolism), hospitalization in intensive care unit, and maternal death [2].

^6^ Neonatal trauma was defined by the existence of at least one of the following criteria: fracture of the clavicle or a long bone, brachial plexus injury, and cephalhematoma [2].

^7^ Severe neonatal morbidity was defined by at least one of the following criteria: 5-minute Apgar score<7, umbilical artery pH < 7.00, need for resuscitation or intubation, neonatal trauma, intraventricular hemorrhage > grade 2, admission to the NICU (neonatal intensive care unit) for>24 hours, convulsions, sepsis, and neonatal death [2].

^8^ Episiotomy complications were defined by the existence of at least one of the following criteria: hematoma, abscess, scar disunion, or required surgery for episiotomy.

FISI: American Society of Colon and Rectal Surgeon’s faecal incontinence severity index [19].

* Student t test

** χ2 test

*** Fisher exact test.

Statistical significance was defined as a *P* value < 0.05.

**Table 2:** Univariate analysis of urinary and anal incontinence at 6 months after midpelvic and low attempted operative vaginal delivery.

	Urinary incontinence			Anal incontinence		
	Yes (N = 196)	No (N = 711)	*P* value	Yes (N = 172)	No (N = 735)	*P* value
**Maternal and labor characteristics**						
Maternal age (years) ^1^	30.0 ± 5.0	28.7 ± 4.7	0.001*	30.5 ± 4.6	28.7 ± 4.7	<0.001*
Multiparity, n (%)	50 (25.5)	159 (24.5)	0.76**	55 (32.0)	149 (22.6)	0.01**
Previous delivery with birth weight > 4000g, n (%)	1 (2.0)	7 (4.5)	0.43***	6 (11.1)	2 (1.3)	0.002***
Weight before pregnancy (kg)^1^	62.9 ± 12.1	61.1 ± 11.1	0.05*	62.3 ± 11.1	61.2 ± 11.2	0.24*
BMI ≥ 30 kg/m^2^ before pregnancy, n (%)	14 (7.1)	36 (5.6)	0.41**	14 (8.2)	32 (4.9)	0.09**
Gestational weight gain >20 kg, n (%)	19 (10.0)	49 (7.8)	0.35**	14 (8.2)	53 (8.3)	0.92**
Antenatal suspicion of macrosomia ^2^, n (%)	9 (4.6)	51 (7.9)	0.12**	19 (11.0)	37 (5.6)	0.01**
Gestational age at delivery (weeks) ^1^	39.5 ± 1.3	39.4 ± 1.4	0.75*	39.5 ± 1.3	39.4 ± 1.4	0.30*
Induced labor, n (%)	35 (17.9)	113 (17.4)	0.88**	42 (24.4)	104 (15.8)	0.008**
Length of labor (min)^1^	388.5 ± 164.4	391.9 ± 168.4	0.81*	390.7 ± 171.2	392.8 ± 166.9	0.88*
2^nd^ stage>3 hours, n (%)	33 (16.8)	111 (17.1)	0.93**	34 (19.8)	109 (16.6)	0.32**
Active phase of 2^nd^ stage > 30 min	76 (38.8)	223 (34.3)	0.25**	67 (39.0)	227 (34.5)	0.27**
Epidural analgesia, n (%)	182 (92.9)	624 (96.1)	0.05**	167 (97.1)	626 (95.1)	0.27**
Persistent occiput position			0.22**			0.24**
Anterior, n (%)	175 (90.2)	574 (88.4)		157 (91.3)	580 (88.4)	
Posterior, n (%)	17 (8.8)	53 (8.2)		9 (5.2)	59 (9.0)	
Transverse, n (%)	2 (1.0)	22 (3.4)		6 (3.5)	17 (2.6)	
ACOG classification			0.26**			0.11**
Mid, n (%)	41 (20.9)	113 (17.4)		24 (14.0)	127 (19.3)	
Low, n (%)	155 (79.1)	537 (82.6)		148 (86.0)	532 (80.7)	
Obstetrician performing delivery			0.33**			0.62**
Senior obstetrician, n (%)	49 (25.0)	182 (28.6)		50 (29.2)	117 (27.4)	
Obstetric registrar, n (%)	147 (75.0)	455 (71.4)		121 (70.8)	470 (72.6)	
Instrument type						
Vacuum, n (%)	50 (25.5)	177 (27.3)	0.63**	39 (22.7)	182 (27.7)	0.19**
Forceps, n (%)	16 (8.2)	36 (5.6)	0.18**	13 (7.6)	38 (5.8)	0.39**
Spatula, n (%)	138 (70.4)	450 (69.3)	0.78**	125 (72.7)	454 (69.0)	0.35**
Sequential use of two instruments, n (%)	8 (4.1)	14 (2.2)	0.14***	6 (3.5)	15 (2.3)	0.37***
Indications for aOVD			0.89**			0.25**
Non-reassuring FHR only, n (%)	81 (41.3)	280 (43.1)		65 (37.8)	289 (43.9)	
Arrested progress only, n (%)	80 (40.8)	264 (40.6)		72 (41.9)	267 (40.5)	
Non-reassuring FHR and arrested progress, n (%)	35 (17.9)	109 (16.8)		35 (20.4)	106 (16.1)	
**Maternal outcome**						
Cesarean delivery after failed operative vaginal delivery, n (%)	2 (1.0)	14 (2.1)	0.31***	3 (1.7)	13 (2.0)	0.85***
Episiotomy, n (%)	170 (86.7)	574 (88.6)	0.48**	155 (90.1)	577 (87.8)	0.40**
3^rd^ or 4^th^-degree perineal lacerations, n (%)	11 (5.6)	15 (2.3)	0.02***	10 (5.8)	15 (2.3)	0.02***
PPH (blood loss>500mL), n (%)	35 (17.8)	117 (18.0)	0.96**	39 (22.7)	109 (16.5)	0.06**
Severe PPH (blood loss>1500 mL), n (%)	4 (2.0)	17 (2.6)	0.65***	6 (3.5)	15 (2.3)	0.37***
Severe maternal morbidity ^3^, n (%)	20 (10.2)	54 (8.3)	0.41**	20 (11.6)	52 (7.9)	0.12**
**Neonatal outcome**						
Birth weight (g) ^1^	3314 ± 396	3322 ± 435	0.81*	3405 ± 420	3300 ± 426	0.004*
Birth weight > 4000 g, n (%)	8 (4.1)	38 (5.9)	0.34**	12 (7.0)	34 (5.2)	0.36**
Cephalic perimeter^1^	34.5 ± 1.3	34.4 ± 1.5	0.54*	34.8 ± 1.5	34.5 ± 1.5	0.003*
Severe neonatal morbidity ^4^, n (%)	14 (7.1)	66 (10.1)	0.21**	14 (8.1)	66 (10.0)	0.46**
**Mid-term registered variables**						
Breastfeeding, n (%)	149 (74.5)	419 (64.6)	0.01**	131 (76.2)	424 (64.4)	0.004*
Episiotomy complications ^5^, n (%)	60 (32.8)	239 (39.5)	0.10**	50 (30.5)	245 (40.0)	0.03*
Pelvic floor muscle training, n (%)	162 (83.1)	500 (77.5)	0.10**	148 (86.6)	504 (77.1)	0.007**
Perineal pain, n (%)	34 (17.7)	79 (12.3)	0.06**	29 (17.1)	83 (12.7)	0.14**
Urinary incontinence, n (%)	196 (100)	0	-	66 (38.4)	130 (19.7)	< 0.001**
Stress urinary incontinence, n (%)	20 (10.5)	-		3 (4.8)	17 (14.4)	0.05***
Urge urinary incontinence, n (%)	62 (32.1)	-		13 (20.3)	49 (38.6)	0.01**
Mixed urinary incontinence, n (%)	115 (58.7)	-		49 (28.5)	66 (10.0)	<0.001**
Difficulty voiding, n (%)	61 (31.9)	-		30 (46.2)	31 (24.6)	0.002**
Severe urinary incontinence, n (%)	2 (1.0)	-		1 (1.6)	1 (0.8)	0.62***
Anal incontinence, n (%)	63 (33.2)	109 (17.0)	<0.001**	172 (100)	0	-
FISI score^1^	12.6 ± 7.2	6.4 ± 5.9	0.02*	12.0 ± 6.7	7.5 ± 4.9	0.04*

Values are crude and adjusted linear regression coefficients (R) with their 95% confidence intervals (CI).

^1^ Values are given as mean ± standard deviation.

^2^ Antenatal suspicion of macrosomia: fundal height measurement at delivery > 37cm and/or ultrasonographic fetal abdominal circumference > 90^th^ p. for gestational age and sex on Hadlock curves [14].

^3^ Severe maternal morbidity was defined by the existence of at least one of the following criteria: third or fourth-degree perineal lacerations, perineal hematomas, cervical laceration, extension of uterine incision at cesarean section, PPH>1500 mL, surgical haemostatic procedure, uterine artery embolization, blood transfusion, infections (endometritis, episiotomy infection, wound infection needed surgery), thromboembolic events (deep vein thrombophlebitis and pulmonary embolism), hospitalization in intensive care unit, and maternal death [2].

^4^ Severe neonatal morbidity was defined by at least one of the following criteria: 5-minute Apgar score<7, umbilical artery pH < 7.00, need for resuscitation or intubation, neonatal trauma, intraventricular hemorrhage > grade 2, admission to the NICU (neonatal intensive care unit) for>24 hours, convulsions, sepsis, and neonatal death [2].

^5^ Episiotomy complications were defined by the existence of at least one of the following criteria: hematoma, abscess, scar disunion, or required surgery for episiotomy.

* Student t test

** χ2 test

*** Fisher exact test.

Statistical significance was defined as a *P* value < 0.05.

In the multiple logistic regression analysis, attempted midpelvic (compared with low pelvic) aOVD was not significantly associated with either symptoms of UI (adjusted odds ratio (aOR) 0.70, 95% CI 0.46–1.05), controlling for maternal age, weight before pregnancy, epidural use, ACOG classification, 3^rd^ or 4^th^-degree perineal lacerations, anal incontinence, and breast-feeding; or symptoms of AI (aOR 1.42, 95% CI 0.85–2.39), controlling for maternal age, parity, body mass index before pregnancy, antenatal suspicion of macrosomia, ACOG classification, 3^rd^ or 4^th^-degree perineal lacerations, cephalic perimeter, breast-feeding, pelvic floor muscle training, episiotomy complications and urinary incontinence (Tables 3 and 4). Third/fourth degree perineal tears were a major risk factor for symptoms of UI (aOR 3.08, 95% CI 1.35–7.00) and symptoms of AI (aOR 3.47, 95% CI 1.43–8.39) 6 months after aOVD. Maternal age over 30 years and breastfeeding were also significant risk factors for symptoms of UI (aOR 1.66, 95% CI 1.19–2.31 and aOR 1.64, 95% CI 1.13–2.38, respectively) and symptoms of AI (aOR 1.81, 95% CI 1.25–2.62 and aOR 1.65, 95% CI 1.09–2.50, respectively) at 6 months postpartum (Tables 3 and 4).

**Table 3:** Multivariate analysis for urinary incontinence at 6 months after midpelvic and low attempted operative vaginal delivery.

Variables	Urinary incontinence (N = 196)	
	Adjusted OR (95% CI)	P *value*
Maternal age > 30 years	1.66 (1.19–2.31)	0.003
Weight before pregnancy > 55 kg	1.73 (1.12–2.66)	0.01
Epidural analgesia	0.52 (0.25–1.06)	0.07
ACOG classification		
Mid	0.70 (0.46–1.05)	0.08
Low	Reference	-
3^rd^ or 4^th^-degree perineal lacerations	3.08 (1.35–7.00)	0.007
Breastfeeding	1.64 (1.13–2.38)	0.009

Values are adjusted odds ratios (OR) with 95% confidence intervals (CI).

Wald tests were performed for testing the significance of the covariates included in the models.

**Table 4:** Multivariate analysis for anal incontinence at 6 months after midpelvic and low attempted operative vaginal delivery.

Variables	Anal incontinence (N = 172)	
	Adjusted OR (95% CI)	P *value*
Maternal age > 30 years	1.81 (1.25–2.62)	0.002
BMI before pregnancy ≥ 30 kg/m^2^	2.29 (1.12–4.71)	0.02
ACOG classification		
Mid	1.42 (0.85–2.39)	0.19
Low	Reference	-
3^rd^ or 4^th^-degree perineal lacerations	3.47 (1.43–8.39)	0.006
Cephalic perimeter > 36 cm	1.74 (1.13–2.65)	0.01
Breastfeeding	1.65 (1.09–2.50)	0.02

Values are adjusted odds ratios (OR) with 95% confidence intervals (CI).

Wald tests were performed for testing the significance of the covariates included in the models.

## Discussion

### Main findings

We investigated the association between the fetal head station, and specifically midpelvic or low pelvic, and pelvic floors disorders, specifically symptoms of UI and AI, 6 months after aOVD, using a prospective based cohort analysis. We found that midpelvic aOVD was not associated with a higher rate of symptoms of UI and AI at 6 months postpartum compared to attempted low pelvic delivery. After multivariate analysis, third/fourth degree perineal tears and maternal age older than 30 years were significant risk factors for symptoms of UI and AI at 6 months postpartum.

### Interpretation

It is difficult to compare our results with the literature because, to our knowledge, previous studies of pelvic floor disorders in the postpartum period after aOVD have never detailed their results according to fetal head station at instrument application [3–5,7,8,11].

Nevertheless, our results are consistent with other well-established findings in the literature about health problems after delivery: frequency of UI around 20% after OVD [23–26], frequency of AI around 20% after OVD [27–32], risk factor for AI (obstetric anal sphincter injury) after OVD [6,30,31,33], perineal tears of third/fourth degree [11,31,33,34], advanced maternal age and pre-pregnancy BMI as major risks factors for UI and AI after delivery [35–39]. We also demonstrated that breastfeeding is a significant risk factor for symptoms of UI and AI at 6 months postpartum. This has previously been demonstrated in a longitudinal cohort study that postpartum incontinence at 24 months was significantly associated with persistent breastfeeding [39], probably explained by hormonal changes [40].

### Strengths and limitations

The principal strength of this study is the use of validated instruments for symptoms of UI, AI and pelvic floor disorders at 6 months postpartum in a large, prospective based cohort study with carefully characterized obstetric patients. This allowed a complete characterization of symptoms in this population. In particular, this is, to our knowledge, the first prospective based cohort study, that directly compares midpelvic and low aOVDs for pelvic floor disorders at 6 months postpartum. Tähtinen et al. [13] noted in a recent systematic review and meta-analysis concerning the long-term impact of mode of delivery on stress urinary incontinence and urgency urinary incontinence that the limitations of their review were largely the weaknesses of the eligible studies with only one randomized trial concerning breech presentation at term [41], and only one prospective cohort [12].

Our study has several limitations. First, as described in detail previously [2], determination of the station of the fetal head and thus classification of the OVDs is relatively subjective and is influenced by fetal head position, molding, and time of assessment (before or after regional analgesia) [1]. Nevertheless, the prevalence of midpelvic aOVD was similar to that in other study [42], and the rates of induced labor, persistent occiput posterior or transverse positions, manual rotation, forceps and spatula, aOVD performed by senior obstetricians and aOVD in an operating room were significantly higher in the midpelvic compared to the low-pelvic aOVD group, as previously shown [2]. This finding suggests that the risk of contamination between the two groups was low [2]. Second, we used the French versions of validated questionnaires which were previously used in published works [8,43], but the French versions of these questionnaires were not validated in French. Third, we reported a relative low rate of respondents (46.7%), but this rate was consistent with others large postpartum evaluations using mailed questionnaire [35,36]. It is plausible that participants declining to respond to a questionnaire at 6 months were at higher risk of anal and/or urinary incontinence than participants included in the study. Nevertheless, the non-respondents differed mainly from the respondents in their rate of neonatal morbidity, a factor that was not related to either symptoms of UI or AI in our study. Four, we reported pelvic floor disorders after midpelvic and low pelvic aOVD at 6 months postpartum without any objective testing regarding urinary leakage or anal incontinence. This time frame in the context of the development of pelvic floor disorders after delivery is considered very short term. Urogynecologic evaluation between 2 and 5 years out from aOVD should be done to be meaningful to patients and physicians [4,35,37].

## Conclusion

We found that neither urinary nor anal incontinence differed at 6 months among women who had midpelvic and low pelvic aOVD. These findings may suggest that midpelvic aOVD should be an alternative valid option to cesarean delivery when the fetus is at midpelvis, because short-term maternal and neonatal morbidity and pelvic morbidity at 6 months are not significantly different between midpelvic and low pelvic aOVD. The data at 6 months postpartum are reassuring and need further studies at long-term to confirm these short-term data.

## Supporting Information

S1 Questionnaire Questionnaire used at 6 months to assess pelvic floor disorders. (DOC)

S1 Table Maternal and labor characteristics and maternal and neonatal outcomes for respondents and non-respondents. (DOC)
