# Supplementary material for: Pelvic Floor Disorders 6 Months after Attempted Operative Vaginal Delivery According to the Fetal Head Station: A Prospective Cohort Study
Source: PLoS One. 2016 Dec 16;11(12):e0168591. doi: 10.1371/journal.pone.0168591 (PMC5161379; doi:10.1371/journal.pone.0168591)
Supplement: S1 Questionnaire — (DOC) [file pone.0168591.s001.doc]

# S1 Questionnaire.

We would like to find out about your pelvic floor symptoms (urinary, perineal, digestive) and we are very grateful that you can help us by filling in this questionnaire. Please answer each question, thinking about the symptoms you have experienced in the last month.

**URINARY SYMPTOMS**

For urinary symptoms, you will see that some questions ask how often you have a symptom:

Occasionally = less than one third of the time

Sometimes = between one and two thirds of the time

Most of the time = more than two thirds of the time

Please put a tick in one box for each question.

1) Do you have involuntary loss of urine? ‭ YES ‭ NO

If yes, you could answer to the following questions:

2) Does urine leak when you are physically active, exert yourself, cough, or sneeze?

‭ Never

‭ Occasionally

‭ Sometimes

‭ Most of the time

‭ All of the time

3) Does urine leak before you go to the toilet?

‭ Never

‭ Occasionally

‭ Sometimes

‭ Most of the time

‭ All of the time

4) Do you have difficulties in emptying your bladder?

‭ Never

‭ Occasionally

‭ Sometimes

‭ Most of the time

‭ All of the time

5) Do you have to rush to the toilet to urinate?

‭ Never

‭ Occasionally

‭ Sometimes

‭ Most of the time

‭ All of the time

6) To what extent have your urinary symptoms affected your ability to perform daily tasks (eg, cleaning, "Do It Yourself", lifting objects)?

‭ Not at all

‭ A little

‭ Somewhat

‭ A lot

7) How often do you leak urine?

‭ Never

‭ Once or less per week

‭ 2-3 times per week

‭ Once per day

‭ Several times per day

8) To what extent do you feel that your life has been spoiled by your urinary symptoms?

‭ Not at all

‭ A little

‭ Somewhat

‭ A lot

**PERINEAL CONSIDERATIONS**

9) Did you performed postpartum pelvic floor exercises? ‭ YES ‭ NO

10) Do you have chronic perineal pain (perineum designates the skin and muscle around the vaginal and anal outlets)? ‭ YES ‭ NO

11) Did you have an episiotomy? ‭ YES ‭ NO

If yes, please answer each question, thinking about the complications concerning your episiotomy you have experienced:

12) Hematoma ‭ YES ‭ NO

13) Abscess ‭ YES ‭ NO

14) Scar disunion ‭ YES ‭ NO

15) Surgery ‭ YES ‭ NO

**DIGESTIVE SYMPTOMS**

We would like to find out about your digestive symptoms and we are very grateful that you can help us by filling in this questionnaire.

16) Do you have involuntary loss of flatus or stool? ‭ YES ‭ NO

17) If yes, for each of the following, please indicate on average how often in the past month you experienced any amount of accidental bowel leakage:

|  | 2 or More Times a Day | One a Day | 2 or More Times a Week | One a Week | 1 to 3 Times a Month | Never |
| --- | --- | --- | --- | --- | --- | --- |
| Gas | ‭ | ‭ | ‭ | ‭ | ‭ | ‭ |
| Mucus | ‭ | ‭ | ‭ | ‭ | ‭ | ‭ |
| Liquid Stool | ‭ | ‭ | ‭ | ‭ | ‭ | ‭ |
| Solid Stool | ‭ | ‭ | ‭ | ‭ | ‭ | ‭ |

*(from Rockwood TH, et al. 1999 & 2004)*

# BREASTFEEDING

18) Did you continue breastfeeding? ‭ YES ‭ NO
